# Analysis of outcomes of laparotomic, laparoscopic, and hysteroscopic symptomatic ısthmocele (niche) repair in Turkish women

**DOI:** 10.1590/1806-9282.20250566

**Published:** 2025-10-27

**Authors:** İnci Halilzade, Elçin İşlek Seçen

**Affiliations:** 1 University of Health Sciences, Ankara City Hospital, Department of Obstetrics and Gynecology – Ankara, Turkey.; 2 Ankara Yıldırım Beyazıt University, Department of Obstetrics and Gynecology – Ankara, Turkey.

**Keywords:** İsthmocele, Niche, Cesarean scar defect, Abnormal uterine bleeding, Fertility

## Abstract

**OBJECTIVE:**

Studies reporting the outcomes of patients after surgical repair of uterine isthmoceles usually have small patient populations. Therefore, the aim of the study was to contribute to the literature by reporting the outcomes of surgical repair of uterine isthmoceles in Turkish women.

**METHODS:**

This retrospective study included 41 patients who underwent surgical repair for symptomatic uterine isthmoceles. The patients were divided into two groups: those who underwent vaginal operative hysteroscopy and those who underwent abdominal laparoscopy and laparotomy.

**RESULTS:**

Surgical repair was performed vaginally in 29 patients (70.7%) using operative hysteroscopy and abdominally (laparotomy and laparoscopy) in 12 patients (29.3%). The isthomocele sac size, mean operative time, and median hospital stay were significantly shorter in the hysteroscopically repaired group (p<0.01, p=0.03, and p<0.01, respectively). Six months after surgery, the rate of persistent isthmocele sac was higher in the hysteroscopically repaired group (p<0.01). The myometrial thickness in the area of the repaired isthmocele sac was thicker in the abdominally repaired group (p<0.01). Among the 12 patients who desired pregnancy and underwent surgical repair, 58.3% (n=7) conceived spontaneously. Of these pregnancies, 71.4% were intrauterine and 28.6% were cesarean scar.

**CONCLUSION:**

Hysteroscopic repair of uterine isthmoceles is advantageous, as it contributes to a shorter operative time and shorter hospital stay. However, complete removal of the isthmocele sac via the abdominal route appears to be more beneficial in terms of live birth rates in future pregnancies. Therefore, we recommend laparoscopic or laparotomic isthmocele repair in patients with fertility desires.

## INTRODUCTION

Uterine isthmocele, also known as the uterine niche, is a scar disorder that occurs due to previous cesarean sections (CS). It is an iatrogenic defect in the myometrium at the site of a previous CS scar due to defective tissue healing. In parallel with the increasing CS rates, the probability of occurrence is 60% in women who have had a CS^1^. In their prospective study, Zampieri et al. reported that 42.3% of patients who had a CS developed an isthmocele^2^.

Patients with isthmoceles are not always symptomatic, but symptoms typically include intermittent abnormal uterine bleeding (AUB), pain, and infertility. Menstrual blood accumulated in the niche cannot be completely expelled due to poor contraction of fibrotic muscle tissue and is gradually emptied. Postmenstrual spotting is the most common symptom (30–55%)^3^. Additionally, scarring associated with future pregnancies leads to obstetric complications such as ectopic pregnancy, abnormal placental implantation, scar dehiscence, and the risk of uterine rupture^4^.

Transvaginal ultrasonography (TVUSG) for the diagnosis of isthmocele is quite valuable due to its diagnostic adequacy, cost-effectiveness, and ease of application^5^. Hysteroscopic (H/S), laparoscopic (L/S), laparotomic (L/T), and vaginal approaches are the methods used in surgical treatment. The method used varies depending on the size of the defect, the presence of symptoms, and childbearing plans^6^. Residual myometrial thickness (RMT) is the vertical distance between the uterine serosa and the apex of the defect, and if the RMT is >3 mm, the H/S approach is recommended. In patients with RMT <3 mm, the L/S or L/T approach is at the forefront due to the risk of uterine rupture^7^.

After treatment, it is necessary to evaluate the regression of symptoms, complications, and obstetric history according to the type of surgery. Studies reporting outcomes of patients after uterine isthmocele repair usually have a small patient population. Therefore, the results are still unclear because the number of patients included in the meta-analyses is insufficient. The aim of our study is to contribute to the literature by reporting the outcomes after surgical repair of uterine isthmocele in Turkish women.

## METHODS

The study included 41 patients aged 18–50 years who underwent surgical repair for symptomatic uterine isthmocele between January 2022 and December 2024 at Ankara City Hospital, a tertiary center. Ethics committee approval was obtained from the Ethics Committee of Ankara City Hospital No. 2 (25-941). This was a retrospective, observational study.

Uterine isthmocele was diagnosed by observing a defect of at least 1 mm depth in the cesarean scar line in the TVUSG report. Isthmocele sac size, depth (height), and longitudinal extent (base) of the isthmocele were measured using the formula: base×height/2. RMT was defined as the shortest visible distance between the uterine serosa and the endometrium in the sagittal plane on TVUSG. Preoperative and postoperative TVUSG were always performed by the same experienced clinician using Voluson E6 equipment (GE Healthcare, Chicago, IL) with a 7.5 MHz vaginal probe. Symptoms related to isthmocele were: pelvic pain, AUB, and secondary infertility. The surgical procedures included H/S, L/S, and L/T. The surgeries were performed by a team of experienced surgeons. In the hysteroscopic procedure, the upper and lower edges of the defect were resected using a cutting ring, and coagulation was performed in the thinnest part of the scar. In laparoscopic and laparotomic procedures, the isthmocele tissue was completely removed by resection from its edges, and the defect was sutured.

Patients were divided into two groups: those who underwent operative hysteroscopy via the vaginal route and those who underwent laparoscopy and laparotomy via the abdominal route. The patients’ ages, number of previous CS, operation duration, operation complications, hospitalization days, preoperative and postoperative 6th-month symptoms, TVUSG findings, postoperative pregnancy status, pregnancy complications, and live birth time were recorded, presented, and compared. Asymptomatic patients and those who did not undergo surgical niche repair were excluded from the study.

Statistical analysis was performed using the Statistical Package for the Social Sciences (SPSS) ver. 21.0 (IBM Corp., Armonk, NY, US). The Kolmogorov-Smirnov test was used to determine the normality of the data. Descriptive parameters were expressed as mean±standard deviation for normally distributed continuous variables and as median (interquartile range) for non-normally distributed continuous variables. Categorical parameters were expressed as numbers and percentages. Independent sample t-tests, Mann-Whitney U tests, and chi-squared tests were used to compare groups. Statistical significance was set at p<0.05.

## RESULTS

The mean age of the 41 patients included in the study was 35.0±6.1 years. There were no patients whose primary reason for admission was secondary infertility or pelvic pain. While, all patients presented with AUB, 12 patients (29.3%) stated that they planned to have children in the future. On ultrasonography, the mean size of the isthmocele sac was 9.8±5.1 mm, and the mean RMT was 3.6±1.1 mm.

**Figure 1:**
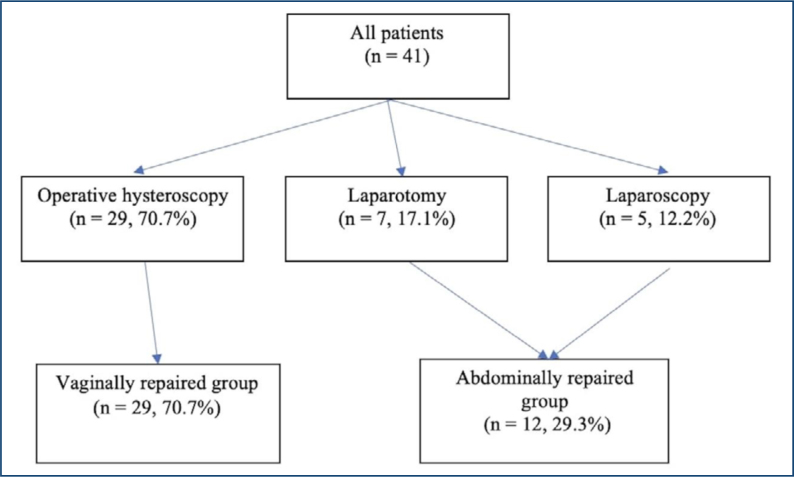
Flow chart of the study population.

Surgical repair was performed vaginally using operative hysteroscopy in 29 (70.7%) patients. In the remaining 12 patients (29.3%), surgery was performed abdominally via laparotomy (n=7, 17.1%) or laparoscopy (n=5, 12.2%) (Figure 1). Surgical complications occurred in two (4.9%) cases, including bladder perforation in one patient who underwent laparoscopic surgery and uterine perforation in one patient who underwent operative hysteroscopy, and these complications were repaired intraoperatively. A comparison of clinical characteristics and preoperative findings of the hysteroscopic and abdominally repaired groups is presented in Table 1.

**Table 1:** Comparison of clinical characteristics and preoperative findings of hysteroscopically and abdominally repaired groups.

Characteristics	Hysteroscopically repaired group (operative hysteroscopy) (n=29, 70.7%)	Abdominally repaired group (laparoscopy and laparotomy) (n=12, 29.3%)	p*
Age (years)	36.3±5.9	31.9±5.5	0.03
Gravidity	3 (1)	2 (2)	0.10
Parity	3 (1)	2 (1)	0.27
Number of previous C/S	3 (1)	2 (1)	0.46
Isthmocele sac size (mm)	8.4±2.7	13.1±7.7	<0.01
RMT (mm)	4.1±0.8	2.3±0.5	<0.01
Operation time (min)	36.9±14.2	100.8±35.5	<0.01
Hospital stays (day)	2 (0)	3 (1)	<0.01

Data is presented as mean±standard deviation, median (interquartile range), or n (%).

* Independent sample t-tests, Mann-Whitney U tests, and chi-squared tests were used to compare groups. C/S: cesarean section; RMT: residual myometrial thickness.

Six months after surgery, 37 of 41 patients (90.2%) attended the control examination, while four patients (9.8%), one from the hysteroscopically repaired group and three from the abdominally repaired group, were lost to follow-up. Twelve of 37 patients (32.4%) complained of persistent AUB. A persistent isthmocele sac was observed in 28 patients (75.7%) on control ultrasonography, and the mean isthmocele sac size was 3.3±1.7 mm. In addition, the mean myometrial thickness in the repaired isthmocele sac location on ultrasonography was 4.2±2.0 mm. A comparison of the postoperative 6th-month postoperative findings of the hysteroscopic and abdominal repair groups is presented in Table 2.

**Table 2:** Comparison of postoperative 6th-month findings of hysteroscopically and abdominally repaired groups.

		Hysteroscopically repaired group (operative hysteroscopy) (n=28, 75.7%)	Abdominally repaired group (laparoscopy and laparotomy) (n=9, 24.3%)	p*
Complaint of AUB				
	No	17 (45.9)	8 (21.6)	0.22
	Yes	11 (29.7)	1 (2.7)	
Persistent isthmocele sac				
	No	2 (5.4)	7 (18.9)	<0.01
	Yes	26 (70.3)	2 (5.4)	
Persistent isthmocele sac size (mm)		5.5±3.5	3.0±1.4	0.04
Myometrial thickness in the repaired isthmocele sac location (mm)		3.5±1.3	6.3±2.3	<0.01

Data is presented as mean±standard deviation or n (%).

* Independent sample t-tests and chi-squared tests were used to compare groups. AUB: abnormal uterine bleeding.

Of the 12 patients who underwent surgical repair and desired pregnancy, 58.3% (n=7) conceived spontaneously. Among these pregnancies, 5 (71.4%) were intrauterine and 2 (28.6%) were cesarean scar. Two patients with scar pregnancies were repaired hysteroscopically, and two were evacuated. The other five pregnancies resulted in a live birth by CS. The mean gestational age was 37.48±0.75 weeks. Uterine isthmocele was repaired by laparotomy in 4 (80%) and hysteroscopy in 1 (20%) of the patients who had a live birth. The mean duration from surgery to achieving pregnancy was 8.4±4.4 months.

## DISCUSSION

In our study, we presented the characteristics, management, and postoperative obstetric outcomes of patients who underwent hysteroscopic, laparoscopic, and laparotomy surgical repair due to symptomatic isthmocele. The operative time and hospital stay were lower in the hysteroscopically repaired group. In the control ultrasonography 6 months after the operation, the rate of persistent isthmocele sac was found to be higher in the hysteroscopically repaired group than in the abdominally repaired group. Myometrial thickness in the area of the repaired isthmocele sac was thicker in the abdominally repaired group. As a result of these, two of the seven women who became pregnant in the postoperative period had scar pregnancies, and five had intrauterine pregnancies and live births. Both scar pregnancies were in the hysteroscopically repaired group.

Due to the increasing incidence of uterine isthmocele, its treatment and subsequent obstetric and gynecological follow-up are important. Although treatment is not recommended in asymptomatic patients, surgical repair of symptomatic isthmocele is an accepted approach in the literature. Hysteroscopy is preferred in patients with RMT>2.5–3 mm^8,9^. In addition, the hysteroscopic technique is the first-line recommended technique due to its shorter operative time and fewer hospital days^10,11^. The effect of the isthmocele sac size on the decision of the type of surgery is also important. In larger sacs, complete removal of the sac via abdominal approach is recommended^4^. Additionally, Smet et al. reported that the combination of hysteroscopic and laparoscopic resection is a good option for the correction of larger isthmoceles to completely remove all fibrotic tissue^12^. In our study, in accordance with the literature, the isthmocele sac was smaller and RMT was higher in patients who underwent hysteroscopic repair compared to patients who underwent abdominal repair. Additionally, our study showed that patients who underwent hysteroscopic repair had shorter operative times and hospital stays.

Studies have shown that there is more than 80% improvement in AUB complaints after isthmocele surgery^4^. AUB recovery rate was reported as 80% in patients undergoing vaginal repair^13^, 71.4% in patients undergoing laparoscopic repair^14^, and 78.6% in patients undergoing hysteroscopic repair^15^. In our study, we determined the overall recovery rate of AUB after surgical repair as 67.6%. The AUB recovery rate was 60.7% in the hysteroscopic repair group and 88.9% in the abdominal surgical repair group. Although a higher recovery rate was seen in the abdominally repaired group, there was no statistically significant difference. In addition, the isthmocele sac was still visible at 6 months postoperatively in the hysteroscopically repaired group, and the myometrial thickness at the repaired isthmocele sac location was thinner. Piriyev et al. reported a 335% increase in postoperative myometrial thickness in patients with isthmoceles who underwent laparoscopic repair. Therefore, they suggested that surgical hysteroscopy was not suitable for this purpose in patients with fertility desire and recommended isthmocele correction by laparoscopic procedure^16^. In contrast, Enderle et al. reported the same recovery rate in patients who underwent hysteroscopic and laparotomic repair in both groups (80%) on ultrasonographic imaging 1-2 months postoperatively^17^. A meta-analysis revealed that there was no significant difference between hysteroscopic and laparoscopic methods in the improvement of postoperative AUB symptoms, but laparoscopic repair had a slight advantage over hysteroscopic repair (78–94% vs. 60–100%)^18^. They also showed that secondary surgery was required in certain patients who only underwent hysteroscopic repair^18^. We believe that this is due to the fact that during hysteroscopic surgery, healthy myometrial tissue is resected from the right and left of the isthmocele sac, thus expanding the size of the damaged myometrial tissue. However, since the damaged tissue was completely removed in the abdominal approach and the normal myometrial tissue was brought to the opposite side and sutured, we believe that we found a lower rate of persistent isthmocele sac and a higher postoperative myometrial thickness.

Another important issue after surgical repair of an isthmocele is the obstetric history. In an isthmocele that has not been treated surgically, there is a risk of uterine rupture in possible pregnancies due to the RMT being thin. In addition, obstetric complications such as scar pregnancy and abnormal placental implantation may occur^4^. Regarding the prognosis of pregnancies that occur after surgical treatment, meta-analyses have small study populations, and more studies are needed on this subject^18,19^. In their meta-analysis, Harjee et al. reported the pregnancy rate after isthmocele surgery in 234 patients as 65.4%. They revealed that 87.1% of these pregnancies resulted in live birth^19^. In our study, the postoperative pregnancy rate was 58.3%, and 71.4% of the pregnancies resulted in live birth. The remaining pregnancies (n=2, 28.6%) were cesarean scar pregnancies, and both were hysteroscopically repaired patients. We attribute this to the fact that hysteroscopic treatment increases the size of the damaged tissue. We also predict that the risk of uterine rupture may increase in possible intrauterine pregnancies after hysteroscopic treatment. In our study, three pregnant women who had a live birth underwent abdominal repair, while one had hysteroscopic repair. The patient who underwent hysteroscopic repair was delivered by CS at 36.2 weeks due to the risk of uterine rupture. The cesarean delivery weeks of the patients who had abdominal repair were 38.0, 38.1, and 37.6 weeks.

The limitation of our study is that none of our patients presented with complaints of secondary infertility or pain. Therefore, we could not provide data on the recovery status of these complaints. However, reporting the pregnancy outcomes of our patients with long-term follow-up is our strength that will contribute to the small number of populations in the literature on this subject.

## CONCLUSION

In conclusion, symptoms improve greatly after surgical repair of a symptomatic isthmocele. Hysteroscopic repair is advantageous because it contributes to symptom improvement, shorter operative time, and a shorter hospital stay. However, the damaged tissue is not removed and is even enlarged. Although some studies report high pregnancy rates and live birth rates after hysteroscopic repair^6^, we believe that complete removal of the damaged tissue, especially for patients with fertility expectations, is more effective in preventing possible obstetric complications and achieving high live birth rates. Therefore, we recommend laparoscopic or laparotomic isthmocele repair with complete removal of the isthmocele sac in patients with fertility desire.

## Data Availability

The datasets generated and/or analyzed during the current study are available from the corresponding author upon reasonable request.
